# TCRpMHCmodels: Structural modelling of TCR-pMHC class I complexes

**DOI:** 10.1038/s41598-019-50932-4

**Published:** 2019-10-10

**Authors:** Kamilla Kjærgaard Jensen, Vasileios Rantos, Emma Christine Jappe, Tobias Hegelund Olsen, Martin Closter Jespersen, Vanessa Jurtz, Leon Eyrich Jessen, Esteban Lanzarotti, Swapnil Mahajan, Bjoern Peters, Morten Nielsen, Paolo Marcatili

**Affiliations:** 1 Department of Bio and Health Informatics, Technical University of Denmark, Kgs. Lyngby, Denmark; 2 Centre for Structural Systems Biology (CSSB), DESY and European Molecular Biology Laboratory, Notkestrasse 85, 22607 Hamburg, Germany; 3 Department of Bioinformatics and Data Mining, Novo Nordisk A/S, 2760 Måløv, Denmark; 4 Instituto de Investigaciones Biotecnológicas, Universidad Nacional de San Martín, Buenos Aires, Argentina; 5 Division of Vaccine Discovery, La Jolla Institute for Allergy and Immunology, La Jolla, CA 92037 USA; 6 University of California San Diego, Department of Medicine, La Jolla, CA 92037 USA; 7 Evaxion Biotech, Bredgade 34E, 1260 Copenhagen, Denmark

**Keywords:** Immunology, Antigen processing and presentation

## Abstract

The interaction between the class I major histocompatibility complex (MHC), the peptide presented by the MHC and the T-cell receptor (TCR) is a key determinant of the cellular immune response. Here, we present TCRpMHCmodels, a method for accurate structural modelling of the TCR-peptide-MHC (TCR-pMHC) complex. This TCR-pMHC modelling pipeline takes as input the amino acid sequence and generates models of the TCR-pMHC complex, with a median Cα RMSD of 2.31 Å. TCRpMHCmodels significantly outperforms TCRFlexDock, a specialised method for docking pMHC and TCR structures. TCRpMHCmodels is simple to use and the modelling pipeline takes, on average, only two minutes. Thanks to its ease of use and high modelling accuracy, we expect TCRpMHCmodels to provide insights into the underlying mechanisms of TCR and pMHC interactions and aid in the development of advanced T-cell-based immunotherapies and rational design of vaccines. The TCRpMHCmodels tool is available at http://www.cbs.dtu.dk/services/TCRpMHCmodels/.

## Introduction

As part of the adaptive immune response, T-cells recognise and kill pathogenic or pathogen-infected cells^1,2^. Understanding the mechanisms of such immune responses is therefore important for the development of cancer immunotherapies and rational vaccine design^3–9^. The activation of T-cell immunity is primarily driven by the interaction between peptides presented by major histocompatibility complexes (pMHCs) and T-cell receptors (TCRs)^1,10,11^. TCRs are found on the surface of T-cells where they recognise protein fragments, named antigens, when these are presented by the MHC on the cell surface of antigen presenting cells. TCRs consist of two membrane-bound chains, which can be either α and β chains or γ and δ chains^12^. The majority of T-cells expresses αβ-TCRs and these T-cells can be further subdivided into cytotoxic T-cells and T-helper cells^13^. Cytotoxic T-cells interact with the MHC class I molecules and are involved in direct killing of pathogen-infected cells, whereas T-helper cells interact with the MHC class II molecules after which they directly or indirectly activate other immune cells to combat the pathogenic infection^14^. In this work, we focus on modelling the TCR-pMHC complex of αβ-TCRs and MHC class I molecules, as these constitute the majority of the available structural complexes.

The TCR-pMHC complex consists of two components, namely the TCR and the pMHC^2^. The MHC class I molecule is a heterodimeric glycoprotein that consists of an α chain and a β2-microglobulin chain. The α chain is composed of three globular domains named α1, α2 and α3 which are highly polymorphic, allowing the MHC variants to accommodate a diverse range of peptides of different lengths and compositions^2^.

Each of the two chains in the αβ-TCR has a variable (V) and constant (C) domain. Located within the variable domains are three complementarity determining region (CDR) loops and these account for the main interaction with the pMHC^15^. The sequence of the CDR loops are determined by a recombination process which leads to a highly diverse set of T-cells with different TCRs^16^. It is assumed that the recombination process can theoretically generate more than 10^15^ T-cell variants^17^, but only a minor fraction of these, 10^6^ to 10^8^, are actually expressed at any given time in the human organism^15^. Despite the high variability in the CDR loop sequence, it has been shown that most CDRs only adopt a limited number of main chain conformations named canonical structures and that these canonical structures can usually be identified by specific sequence features^18–20^.

In the past, numerous sequence- and structure-based tools have been developed to predict and model the structure of and/or the interaction between the peptide and the MHC class I molecule^21–27^. Several structure-based tools for modelling the TCR have likewise been developed in the past^18,28^. In recent years, there has been an increased focus on the TCR-pMHC binding accompanied by the development of tools for predicting the interaction between the pMHC and the TCR^29–32^. In particular, previous work has demonstrated how a simple force-field-based approach can be used to identify the cognate pMHC target of a TCR given the availability of structural models of the TCR-pMHC complex^33^. Additionally, structural models have been used to analyse how mutations in the peptide affect the binding to a specific TCR^34^. While tools to deal with peptide-MHC binding and predicting T-cell epitopes have been developed over the last decade^14–17^, limited work has been dedicated to the task of generating accurate TCR-pMHC models. In order to aid this development, we present a novel framework for automated modelling of TCR-pMHC complexes. The modelling pipeline, named TCRpMHCmodels, utilises the amino acid sequences of the MHC, peptide and TCR α and β chains. In a fully automated manner, the pipeline applies a series of simple comparative modelling steps to construct structural models of the pMHC, the TCR, and, subsequently, the TCR-pMHC complex. The tool does not include any assessment of the binding energy or prediction and ranking of potential T-cell epitopes. However, we believe that the models produced by our tool in combination with refined binding energies can be used to provide valuable insights into the mechanisms underlying the interaction between TCRs and pMHCs, and that the models can guide the refined prediction of T-cell epitopes.

Here, we report the large-scale benchmark evaluation of different modelling strategies, including single- versus multi-template modelling, as well as different similarity measures for optimal template selection, to arrive at the optimal method implemented in TCRpMHCmodels. The performance of TCRpMHCmodels is benchmarked against TCRFlexDock^29^, a specialised protein docking method for identifying the correct orientation between the TCR and pMHC structure. Lastly, we test the performance of TCRpMHCmodels on 14 TCR-pMHC structures deposited in the Protein Data Bank (PDB)^35^ after the generation of the TCR-pMHC template database.

## Results

TCRpMHCmodels is an automated modelling pipeline for generating structural models of a TCR-pMHC complex using only the amino acid sequence as input. This method adopts a template-based modelling approach, generating a structural model of a given protein sequence (target), using one or more experimentally determined structures of related homologous proteins (templates).

**Figure 1:**
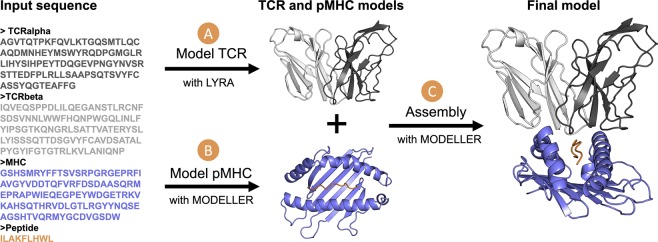
Flowchart of the computational framework for modelling TCR-pMHC complexes, from the input sequence to the final TCR-pMHC model. The MHC molecule is depicted in blue and the peptide in orange, while the two chains of TCR, α and β, are represented in light and dark grey, respectively.

The initial steps in the TCRpMHCmodels pipeline involves the modelling of the TCR and the pMHC separately. These two models are then combined when building the final TCR-pMHC complex. The TCR is generated with LYRA^18^, using templates from a TCR database (see Method section), while the pMHC is generated with MODELLER^36^, using templates from a pMHC database (see Method section). The full TCR-pMHC model is then generated with MODELLER using the TCR and pMHC model as templates together with one or more templates from the TCR-pMHC database (see Method section, Fig. 1).

MODELLER is a comparative modelling tool for predicting the three-dimensional structure of proteins^37^. The tool needs an initial alignment of the sequence to be modelled and one or more known structures. Based on the alignment, MODELLER automatically extracts spatial features, such as Cα-Cα distances, hydrogen bonds, and main chain and side chain dihedral angles, and transfers these from the templates to the target. Lastly, the three-dimensional model is obtained by satisfying all spatial restraints as accurately as possible.

LYRA is a tool that can predict the structure of TCRs. The tool starts by selecting the best framework template for each chain in the TCR, after which it uses the canonical structure model to select the best templates for each of the CDRs. The CDRs are then grafted onto the framework templates which is then merged and the side chains are repacked to generate the final TCR model.

To ensure good model quality of the TCR-pMHC complex, we have optimised each of the steps in TCRpMHCmodels. The results from these optimisations are described in the following sections. All RMSDs, unless otherwise specified, are calculated on Cα atoms only.

### pMHC model optimisation

The first step in the TCRpMHCmodels method is building a structural model of the pMHC. In order to assess the model quality of this step, we have generated structural models for each structure in the pMHC database using a leave-one-out (LOO) approach and evaluated the quality of the generated models by comparing them to their native structure found in the pMHC database. For the model optimisation process, we imposed four different template-target sequence identity thresholds of 99.9%, 95%, 90% and 80%, selecting only structural templates with a sequence identity below the given threshold (see the Method section). By using different sequence identity thresholds, we thereby generate a more diverse set of structural models with both high and low sequence identity to the template database.

**Figure 2:**
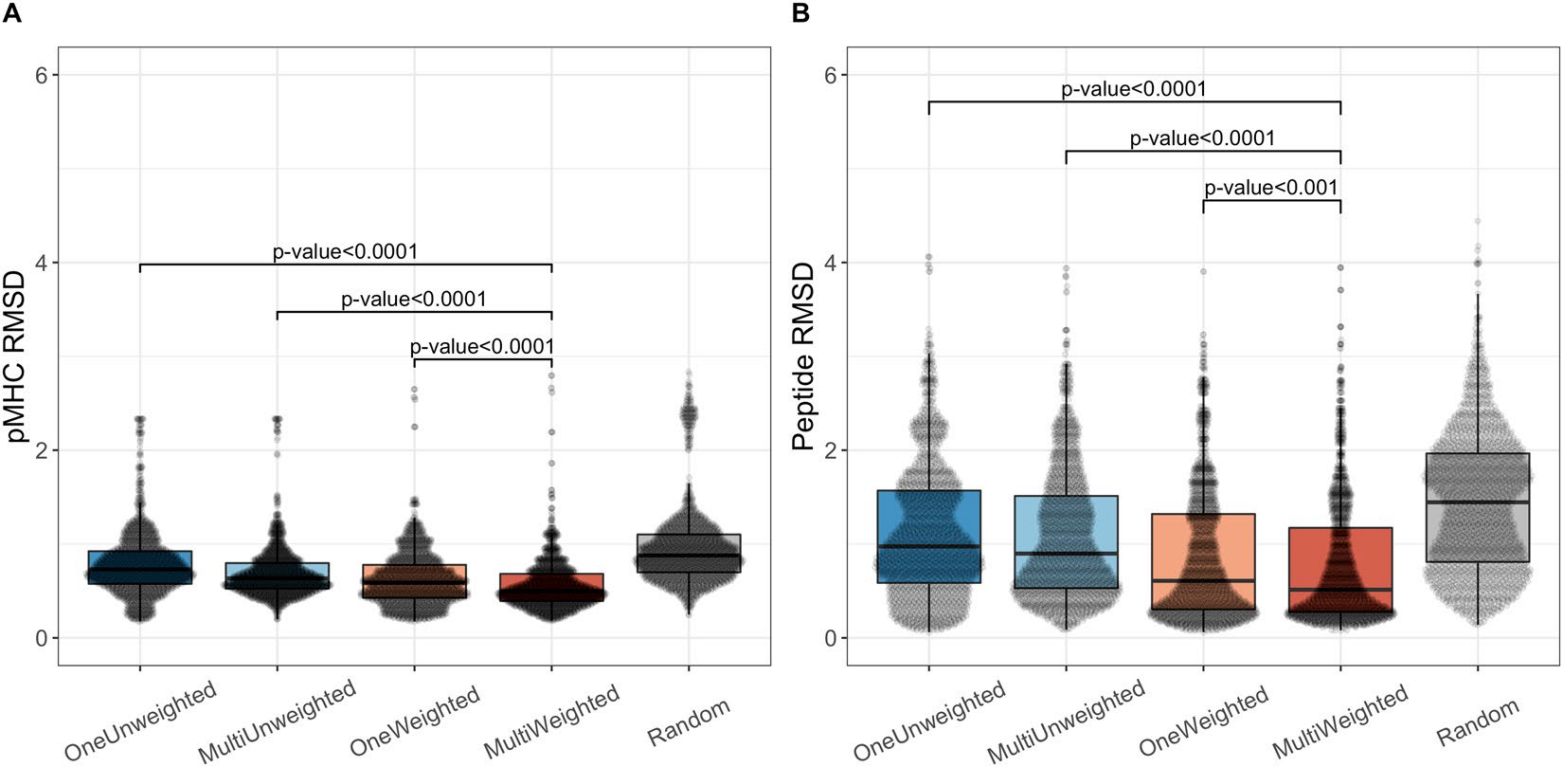
RMSD accuracy for the different template selection methods. (**A**) The RMSD for the pMHC complex. (**B**) The RMSD for the peptide. For each target in the template database, we generate four models using the four different sequence identity thresholds. The OneUnweighted method uses only a single pMHC template with no weights on the sequence identity. The MultiUnweighted also have no weights on the sequence identity but this method uses multiple templates. The OneWeighted method uses only a single pMHC template and a weighted sequence identity. The MultiWeighted method uses the weighted sequence identity and multiple templates. The four different template selection methods are compared with a random baseline (see the Method section for more details). The p-values were obtained using the Wilcoxon signed-rank test.

Using the LOO approach with the four sequence identity thresholds, we generated four structural models for each target in the pMHC database. When modelling the pMHC, we also investigated four different template selection methods, which we denote OneWeighted, OneUnweighted, MultiWeighted and MultiUnweighted, to evaluate the effect of using a single or multiple templates as well as using an unweighted or weighted sequence identity score for template selection. The four different template selection methods were further compared with a random baseline. For more details on the template selection methods and the random baseline (see the Method section). The results of this analysis are illustrated in Fig. 2 and Supplementary Fig. S2.

From Fig. 2A,B, we observe that the MultiWeighted method performed significantly better than the other methods, both when comparing the RMSD of the pMHC and the peptide. The median RMSD values of the pMHC and the peptide in the MultiWeighted method are 0.54 Å and 0.50 Å, respectively. For comparison, the median RMSD values of the Random method are 0.88 Å and 1.44 Å for the pMHC and the peptide, respectively. The improved accuracy of the peptide RMSD shows that the MultiWeighted method is capable of accurately modelling this part of the pMHC complex, which is less conserved and fundamental for the TCR specificity. Similar conclusions were obtained using the TM-score (see Supplementary Fig. S1). Due to the improved accuracy, we therefore selected the MultiWeighted method as the default method for building the pMHC in TCRpMHCmodels.

**Figure 3:**
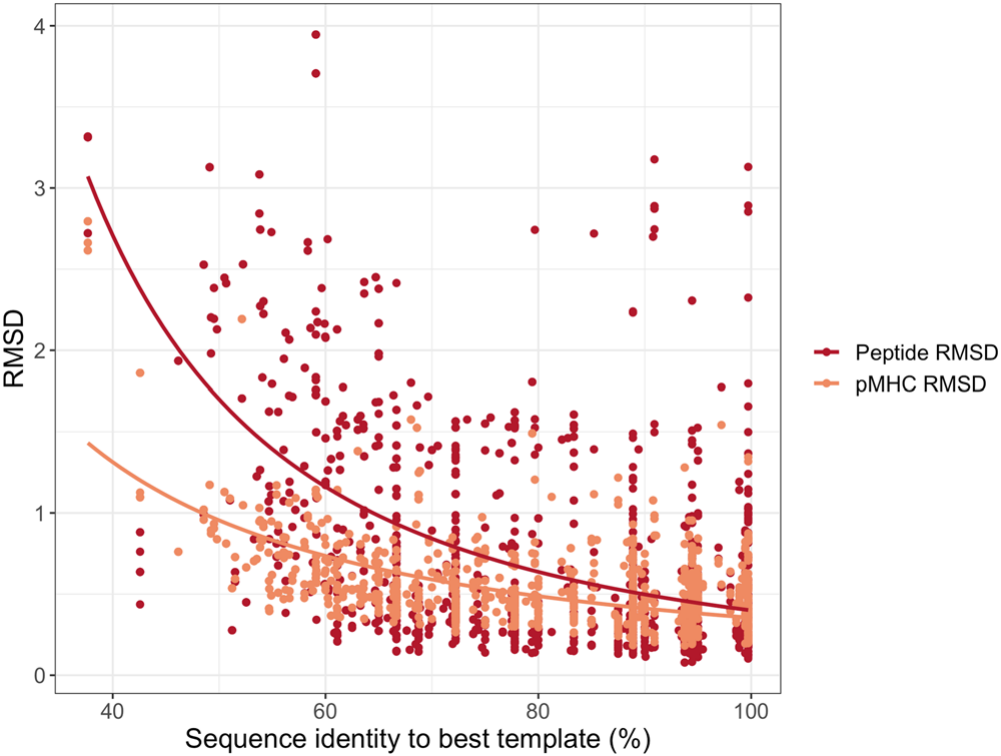
Benchmark results for the pMHC models generated using the MultiWeighted method. Chothia-Lesk plot showing the RMSD accuracy for the pMHC (orange) and the peptide (red) against the sequence identity to the best template.

In Fig. 3, we display the accuracy of the MultiWeighted method in a more detailed manner, showing how the modelling accuracy of the pMHC and the peptide depends on the sequence identity to the best template, using a Chothia-Lesk plot^38^. From this figure, it is clear that the modelling accuracy for the pMHC complex is in general very high (less than 1 Å), even in situations where the best template shares very limited similarity to the target. However, it is also clear that this high accuracy is driven by the very conserved structure of the MHC, and that the picture is very different when focusing only on the peptide. To further investigate this, we analysed how the pMHC model accuracy depends on the peptide length and the sequence identity, see Supplementary Fig. S3 and S4 for details. Supplementary Fig. S3 demonstrates (as expected) that longer peptides tend to have higher peptide RMSDs. The same tendency is observed when investigating models generated using different sequence identity thresholds for template selection, see Supplementary Fig. S4.

### TCR model accuracy

The TCR subunit of the TCR-pMHC complex is modelled using LYRA^18^. LYRA is an automated method for modelling TCRs and it generates models of high accuracy with a 1.48 Å global RMSD and 2.13 Å binding site RMSD. The main advantage of LYRA is that it uses the so-called canonical structure method to select the best templates for the CDR loops. The canonical structures are conserved and limited in conformations of CDR loops that can usually be identified by sequence-based rules. The canonical structure model has been proven valid for both antibodies and TCRs^18–20^ and LYRA is the only automated method that uses the canonical structure method for building structural models of the TCR.

### TCR-pMHC model optimisation

The final task of TCRpMHCmodels is to find the optimal approach for assembling the TCR and pMHC model to form the TCR-pMHC complex. In our TCR-pMHC modelling pipeline, this is achieved with MODELLER using the TCR and pMHC model as templates together with one or more templates from the TCR-pMHC database.

In order to assess the model quality of this step, we generated structural models for each structure in the TCR-pMHC database using a LOO approach, and we then evaluated the quality of the generated models by comparing them to their native structure found in the TCR-pMHC database.

**Figure 4:**
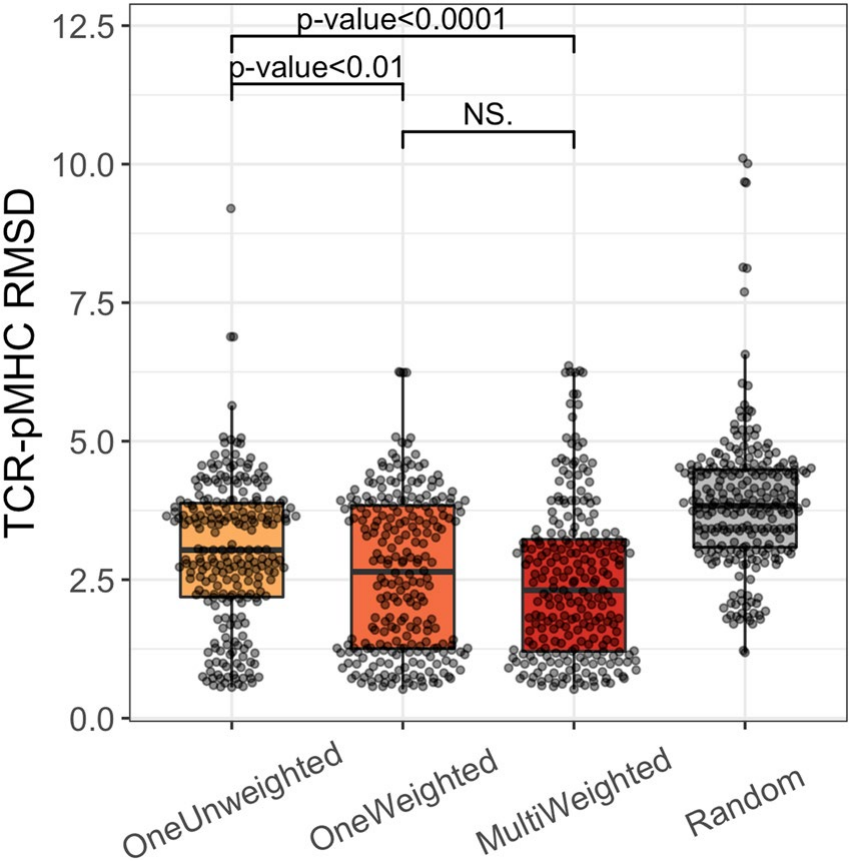
The TCR-pMHC RMSD accuracy for the different template selection methods. For each target in the template database we generated four models using the four different sequence identity thresholds and evaluated the generated models using the RMSD for the TCR-pMHC complex. The OneUnweighted method uses only a single TCR-pMHC template with no weights on the sequence identity. The OneWeighted method uses only a single TCR-pMHC template and a weighted sequence identity. The MultiWeighted method uses the weighted sequence identity and multiple templates. The three different template selection methods are compared with a random baseline shown in grey. The p-values were obtained using the Wilcoxon signed-rank test. The RMSD accuracy of the TCR, the pMHC and the peptide are shown in Supplementary Fig. S5.

Using the LOO approach with the four different sequence identity thresholds, we then generated four models for each target in the TCR-pMHC database. When modelling the TCR-pMHC, we investigated three different template selection methods, which we denote OneUnweighted, OneWeighted and MultiWeighted, to evaluate the effect of using a single or multiple templates as well as using an unweighted or weighted sequence identity score for template selection. The results from this analysis are depicted in Fig. 4.

From Fig. 4, we observe that the MultiWeighted method has a lower median than the other methods and we therefore used this method as the default template selection method in the final TCRpMHCmodels pipeline. We also show the TM-scores (see Supplementary Fig. S6) and the Chochia-Lesk plot (see Supplementary Fig. S7). The MultiWeighted method has a median TCR-pMHC RMSD of 2.31 Å which shows that model accuracy for the TCR-pMHC complex is in general very high. Comparing the median TCR-pMHC RMSD from the MultiWeighted method with the median RMSD of the Random method, we see an 86% improvement in the accuracy.

**Figure 5:**
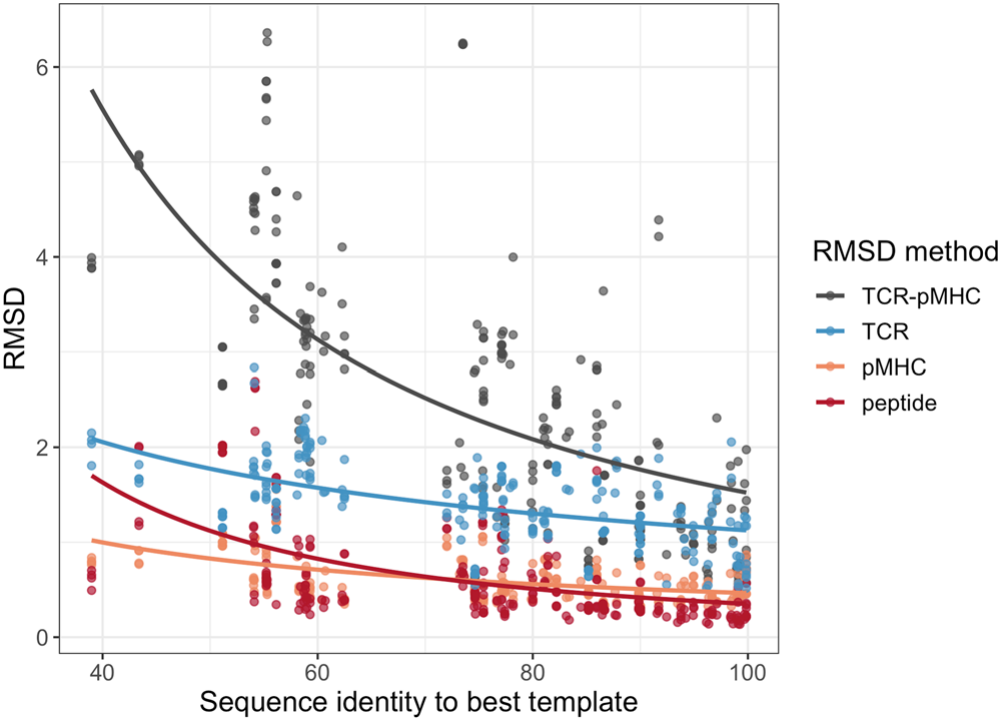
RMSD accuracy for the TCR-pMHC models generated using the MultiWeighted method. Chothia-Lesk plot showing the RMSD accuracy for the TCR-pMHC (grey), the TCR (blue), the pMHC (orange) and the peptide (red) against the sequence identity to the best template.

Figure 5 shows the accuracy of the MultiWeighted method in a more detailed manner, by plotting the model accuracy based on the sequence identity to the best template, using a Chothia-Lesk plot.

From Fig. 5 we see that the modelling of final TCR-pMHC complexes is much more dependent on the sequence identity to the templates compared to the pMHC and the TCR subunits. This could be explained by the fact that the conformation of the TCR-pMHC is much more variable than the conformation of the TCR or the pMHC alone.

### Benchmark against TCRFlexDock

Our optimised TCRpMHCmodels pipeline was benchmarked against the TCRFlexDock method, a specialised protein docking method for finding the correct orientation between the TCR and pMHC to form the final TCR-pMHC complex (see the Method section). The TCRFlexDock protocol applies a set of iterative Monte Carlo moves and side chain packing, combined with refinement of both peptide and CDR loop conformations^29^. The TCRFlexDock docking protocol was run 1000 times to generate 1000 TCR-pMHC models, which were then scored using ZRANK^39^ to select the best models.

**Figure 6:**
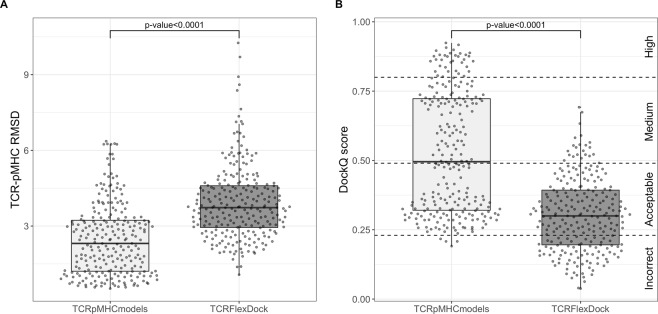
Benchmark analysis of the TCR-pMHC models. (**A**) Shows the TCR-pMHC RMSD accuracy between the models produced by TCRpMHCmodels and TCRFlexDock. (**B**) Shows the DockQ scores between the models produced by TCRpMHCmodels and TCRFlexDock. The statistical comparison was performed using the Wilcoxon signed-rank test.

To compare the quality of the models produced by the two different methods we used both RMSDs and DockQ scores^40^. The result of the benchmark analysis is shown in Fig. 6.

We compared the accuracy of the models produced with the TCRpMHCmodels pipeline and the TCRFlexDock protocol, using RMSDs and DockQ scores (see Fig. 6). The RMSD is a measure of the average distance between the Cα atoms from the model and the Cα atoms in the native structure solved using X-ray crystallography. This measure, while accounting for the overall accuracy of the model, does not take into account side-chain placement which is critical for identifying molecular interactions and the TCR-pMHC interface as a whole. This is partially accounted for by using the DockQ score, a model quality measure derived by combining Fnat, LRMS, and iRMS, three measures of model quality proposed and standardised by the Critical Assessment of PRedicted Interactions (CAPRI) community^41^. Fnat is the fraction of native and non-native residue-residue contacts in the interface, LRMS is the RMSD of the backbone atoms in the ligand after superimposing only the receptor from the native and non-native structure, and iRMS is the backbone atoms of all interface residues^42^. The DockQ score ranges from 0 to 1 and can be used to assign the quality of a model into the four classes: Incorrect (DockQ score < 0.23), Acceptable (DockQ score ≥ 0.23 and DockQ score < 0.49), Medium (DockQ score ≥ 0.49 and DockQ score < 0.80) and High (DockQ score ≥ 0.80)^40^. From Fig. 6, we observe that the models generated with TCRpMHCmodels were significantly more accurate than the models generated using the TCRFlexDock protocol, both in terms of RMSDs and DockQ scores. The median RMSD values of TCRpMHCmodels and TCRFlexDock were 2.31 Å and 3.73 Å, respectively, while the median DockQ scores were 0.50 and 0.3, respectively. Looking only at the DockQ scores we see that almost all the models produced by the TCRpMHCmodels pipeline had an acceptable, medium or high model quality, whereas the models produced using the TCRFlexDock protocol had an incorrect, medium or acceptable model quality. The model quality measure Fnat, LRMS, and iRMS is shown in Supplementary Fig. S8. This indicates that the TCRFlexDock protocol is less accurate at identifying the correct conformation of the TCR-pMHC complex compared to TCRpMHCmodels, even though the TCRFlexDock protocol optimises both the TCR orientation, the CDR loop conformation and the MHC bound peptide conformation during docking.

To better understand the quality of the models generated by TCRpMHCmodels and TCRFlexDock, we made a visual inspection of the models with the highest and lowest quality for each method (see Supplementary Fig. S9). We here observe that the models produced by TCRpMHCmodels are better at predicting the interface between the TCR and pMHC compared to TCRFlexDock. The model with the lowest quality from TCRpMHCmodes had a Fnat score of 0.38, indicating that around 38% of the native residue-residue contacts in the interface where correctly predicted. The model with the lowest quality from TCRFlexDock had Fnat score of 0.02. This low Fnat score would be classified as an incorrectly predicted interface as only around 2% of the native residue-residue contacts in the interface where correctly predicted. Looking at this low-quality model generated by TCRFlexDock, it can be observed that the CDR loops of the TCR is mainly interacting with one of the sides in the MHC molecule instead of interacting with the peptide as would be expected (see Supplementary Fig. S9).

**Figure 7:**
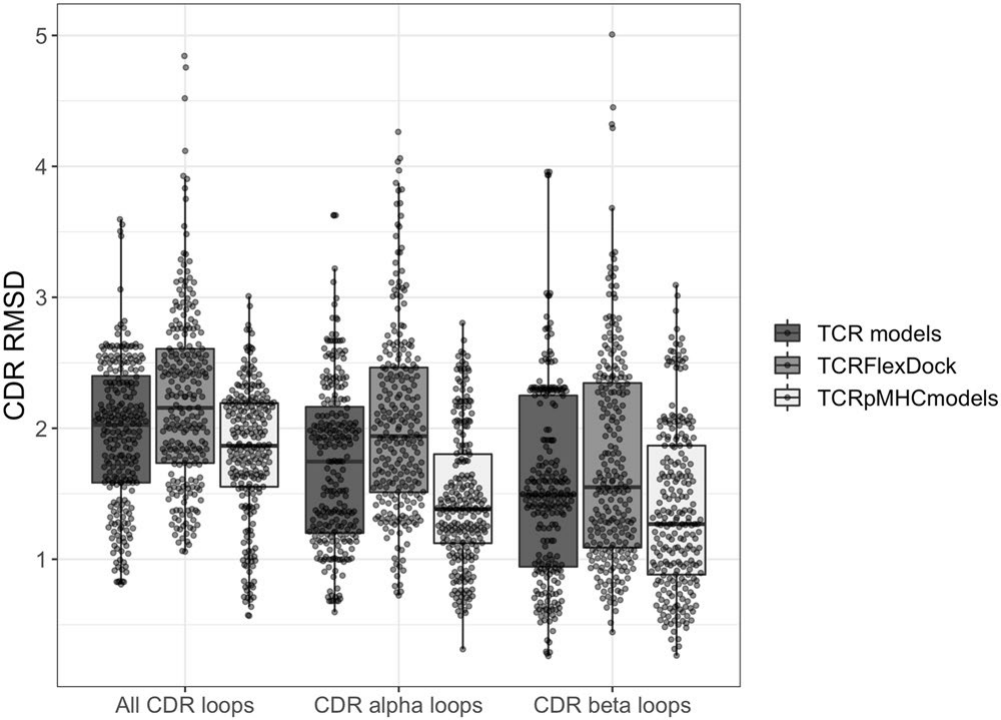
CDR accuracy of the TCR-pMHC models from the benchmark analysis. Shows the CDR RMSD accuracy between the TCR-pMHC models produced by the TCRpMHCmodels pipeline and TCRFlexDock protocol, compared to the initial TCR model produced by the TCR-pMHC pipeline.

We further investigated the CDR loop accuracy between the models generated by TCRFlexDock and TCRpMHCmodels and compared these to the initial TCR model produced by LYRA (see Fig. 7). Looking only at the RMSD for the CDR loops, we observe that the models generated with TCRpMHCmodels have a slightly better loop accuracy compared to the initial TCR models. Generating the final TCR-pMHC complex must therefore change the loop conformation of the CDRs to better fit the peptide-MHC, thereby generating CDR loops which are closer to the loops found in the native TCR-pMHC complex. In comparison to TCRpMHCmodels, the CDR loop accuracy of the model generated with TCRFlexDock decreases, both compared to the initial TCR model and the models generated with TCRpMHCmodels.

### Benchmark against new structures

TCRpMHCmodels was benchmarked using 14 TCR-pMHC structures deposited in IEDB after the TCR-pMHC template database was created. Note that 4 additional structures were available, that could not be modelled; two due to lack of available CDR templates and two due to lack of available TCR-pMHC templates with the correct peptide length. For each of the 14 cases, we generated a single model using TCRpMHCmodels. The average RMSDs for the TCR-pMHC, TCR, pMHC and peptide were 3.20 Å, 1.81 Å, 0.69 Å and 0.77 Å, respectively. For more details see Supplementary Fig. S10 and Supplementary Table S1. This data suggests that TCRpMHCmodels generates accurate models for both the TCR and pMHC complex but is less accurate at predicting the TCR orientation over the pMHC and these predictions should therefore not be over-interpreted. The model accuracy for these new structures is comparable to the results shown in Fig. 5, with the exception of one structure (PDB ID: 5TEZ^43^). The 5TEZ complex has a high sequence identity of 81% to the best TCR-pMHC template (PDB ID: 5EUO), but the resulting model has a relatively poor accuracy (TCR-pMHC RMSD = 5.66 Å). The 5EUO and 5TEZ are both complexes of TCRs bound to the HLA-A2-restricted Influenza A GIL peptides, and hence share 100% identity to the peptide and the MHC. However, the two TCRs are very different (sequence identity of 37% for the α chain and 52% for the β chain), resulting in the TCR of 5TEZ adopting a non-canonical binding orientation to the pMHC^43^. Modelling the 5TEZ structure is, therefore, a highly challenging case, as there are no good templates found in our template database.

## Discussion

Here, we present TCRpMHCmodels, an automated pipeline for building structural models of TCR-pMHC complexes. Using as input only the amino acid sequence of a target TCR-pMHC, TCRpMHCmodels automatically identifies the best structural templates, generates the best target-template sequence alignment and builds a structural model of the target using comparative modelling. The structural models have a high quality and are generated within a computational time of only 2 minutes.

It has been suggested that using multiple templates can increase the model accuracy for comparative models^44^, especially when modelling protein complexes with multiple chains^45,46^. Using multiple templates is harder than it appears, since finding the optimal combination of templates is non-trivial^47^. Including all suitable template candidates usually leads to accumulation of noise and wrong templates which decreases the model quality^33,46^. However, each additional template increases the probability of detecting a template with the correct structural conformation. Finding the right balance is therefore very important when using multiple templates.

In this study, we have evaluated different template selection methods, including single- versus multi-template modelling. Comparing single- versus multi-template modelling of the TCR-pMHC complex, we found that using multiple templates produced the most accurate models. In our multiple template selection method, we always included the template with highest sequence identity; additional templates were added if they had an identity of less than 95% to any template already selected and their identity to the target was at least 80% of the identity of the best scoring template. By doing this, we ended up with a non-redundant list of templates which were then used for the multi-template modelling. This both decreases the number of templates used and increases the chance of selecting structures with the correct conformation.

In the present study, we evaluated the effect of using a weighted sequence identity score by changing the weight of the different chains in the TCR-pMHC complex. We here showed that this weighted identity score achieved the best model accuracy, both when modelling the pMHC and the TCR-pMHC complex.

TCRpMHCmodels first models the pMHC and the TCR separately, after which these are assembled in an additional modelling step to form the full TCR-pMHC complex. The reasoning for modelling the TCR and the pMHC as separate units is that there are more structures of the TCR and the pMHC as separate units, than for the full TCR-pMHC complex. By modelling the TCR and the pMHC separately, we have a larger number of templates which can be used in the comparative modelling step, resulting in more accurate models. This is especially true when modelling the CDR loops in the TCR and the MHC bound peptide, as these parts are more variable and therefore more difficult to model. In the final modelling step, the two models of the TCR and pMHC are used as templates, together with one or more templates of the full TCR-pMHC complex. Using this additional modelling step is a simple way of assembling the TCR and pMHC, and we here show that this approach gives more accurate models than using the more traditional docking approach.

We compared TCRpMHCmodels with TCRFlexDock^29^ and showed that TCRpMHCmodels significantly outperformed TCRFlexDock, both at predicting the full TCR-pMHC complex, the TCR-pMHC interface and the CDR loop conformations. The two methods use a different approach for modelling the TCR-pMHC complex. TCRFlexDock uses a flexible backbone docking protocol based on RosettaDock^48^ to perform TCR-pMHC docking and uses ZRANK^39^ to identify the best TCR-pMHC complexes. The TCRFlexDock protocol was optimised using structures of crystallized TCR and pMHC complexes for which a crystallized structure of the full TCR-pMHC complex also existed. After optimising the TCRFlexDock protocol, the authors of TCRFlexDock then show how the protocol can also produce accurate TCR-pMHC complexes using TCR and pMHC models instead of crystallized structures. TCRpMHCmodels on the other hand is based on a comparative modelling approach and no explicit docking is performed. To make our tool accessible we have implemented TCRpMHCmodels into a web server, which is both fast and easy to use. In contrast to this, the TCRFlexDock protocol is not readily implemented or available as a web server and is both time and computationally intensive as it takes on average 130 CPU hours to run the complete protocol on a single complex. Finally, as the authors of the TCRFlexDock method mention, using TCR and pMHC models is more challenging than using crystal structures, so one reason for the relatively low accuracy of TCRFlexDock in this study could be that we have here only used TCR and pMHC models rather than crystal structures as the initial input for the TCRFlexDock protocol.

A key factor in determining the accuracy of TCRpMHCmodels is the availability of templates suitable for the comparative modelling steps. The current implementation of TCRpMHCmodels is limited to model structures where the length of the bound peptide matches the length of the structures in the pMHC and TCR-pMHC template databases. In practice, this limits the application of the current tool to only model structures with 8–11mer peptides bound in the peptide-binding groove. Also, the accuracy of the tool was demonstrated (as is the case for all comparative modelling approaches) to depend strongly on the sequence identity between the target entry and the template used for modelling. Due to the availability of only a relatively small number of known structures, this dependency has the most pronounced effect when it comes to the full TCR-pMHC template database. This was demonstrated in the case of the 5TEZ PDB structure, where TCRpMHCmodels was shown to achieve an unexpected low predictive performance imposed by the lack of a suitable TCR-pMHC template sharing the non-canonical TCR binding orientation of 5TEZ. This problem could potentially be resolved in the future by including new TCR-pMHC structures into our internal template database as soon as these are deposited into IEDB (https://www.iedb.org/).

It has been shown that structural features of the pMHC complex can shape the TCR repertoire, indicating that key features for TCR recognition may come from the combined structure of the pMHC complex^49^. Furthermore, it is known that a given TCR has the potential to recognise different pMHC complexes, in a process known as T-cell cross-reactivity^49^. Understanding T-cell cross-reactivity is very important for TCR-based immunotherapies, as cross-reactive T-cells can cause serious or even fatal side effects^50,51^. Unfortunately, the available structural data for cross-reactive TCRs and pMHCs is not large enough to draw any conclusions on the ability of our tool to model such cases, but as an illustrative example, we have included the modelling of some cross-reactive peptides and TCRs in Supplementary Figs S11 and S12. In all cases, the accuracy of the models is similar to or marginally worse than the average accuracy of the tool. Future work regarding integration of structural modelling of the TCR-pMHC interaction interface with refined binding energy models might aid in defining such cross-reactivities and allow the development of corresponding predictive models.

Here, we have shown that TCRpMHCmodels generates accurate structural models of the TCR-pMHC complex and that it outperforms TCRFlexDock, a specialised docking protocol for assembling TCR and pMHC molecules. We believe that this work has generated the foundation for future work within the prediction of TCR-pMHC interactions, and we expect the model performance to increase as more structural and sequence data describing TCR-pMHC interactions becomes available.

## Method

### Template databases

TCRpMHCmodels applies three structural databases which are used for modelling the pMHC, the TCR, and the complete TCR-pMHC complex, respectively. At each step, one or more templates are selected from each database according to their sequence identity. In the sections below, we describe the generation of these structural template databases.

### The pMHC database

The pMHC database included 455 non-redundant pMHC structures. The structures found in the database were identified using the Immune Epitope Database (IEDB)^52–54^, with a few additional pMHC structures from the Protein Data Bank (PDB)^35^. Using the sequence from these structures we then generated an in-house Hidden Markov Model (HMM) profile for the MHC class I chain. The in-house HMM was generated using the HMMER software (version 3.1) http://hmmer.org/ with the HMM profile from Pfam^55^ called MHC_I.hmm (accession number: PF00129). This HMM profile includes the α1 and α2 domains of the MHC class I family. We first used hmmsearch to identify PDB entries with a sequence that matched the HMM profile obtained from Pfam. To remove false positive “hits”, we used an E-value threshold of 10^−5^ and only selected entries with a full sequence bit score larger than 250. This yielded 700 PDB entries. All identified entries were then aligned to the MHC class I HMM profile from Pfam using hmmalign to generate a multiple sequence alignment (MSA). We here included the options–trim to exclude residues at the protein terminals that did not fit the HMM model. By performing a manual analysis of the MHC molecules in the database, we found that some of the entries included uncommon insertions at specific positions. These few insertions were primarily found in chicken and canine and, to include these in the HMM, we therefore constructed an in-house HMM profile matching all the identified entries. This new HMM profile was made using hmmbuild using the–symfrac 0 option. The resulting HMM profile contained 181 positions and included all the uncommon insertions, and it identified the same set of pMHC molecules as the original Pfam profile.

The database was next cleaned up by removing pMHC structures without a peptide or with missing residues in the peptide. This reduced the database to 645 pMHC structures. We then used CD-HIT^56^ with a global sequence similarity threshold of 100% to ensure that that the final database only contained unique pMHC structures.

### The TCR database

The TCR database was obtained from LYRA^18^. This database consisted of 105 paired TCR chains, two individual α chains and nine individual β chain structures. For more details see^18^.

### The TCR-pMHC database

The TCR-pMHC database included 61 non-redundant TCR-pMHC structures. These were identified using IEDB^52–54^, with a few additional structures from the Protein Data Bank (PDB). The additional TCR-pMHC structures were found by aligning each entry in the PDB database to the in-house MHC class I HMM profile, plus the HMM profile for the TCR α and β chain. We then used PISCES server^57^ on all the identified TCR-pMHC structures to exclude redundant entries and to remove structures with a resolution above 3 Å. Furthermore, we removed PDB structures with missing residues in the peptide.

**Figure 8:**
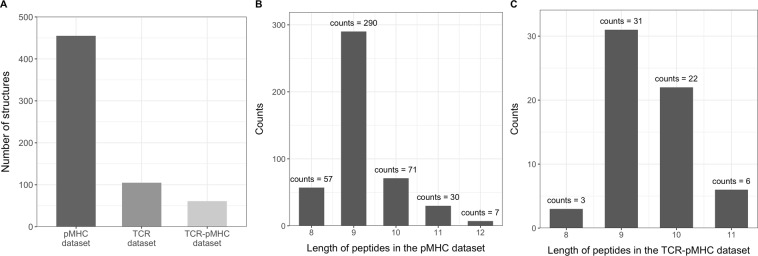
Database visualization. (**A**) Number of structures in each template database. (**B**) The peptide length distribution of structures in the pMHC database. (**C**) The peptide length distribution of structures in the TCR-pMHC database.

An overview of the different databases is shown in Fig. 8. From Fig. 8A, we observe that the pMHC database contains the largest amount of structures, followed by the TCR database and lastly the TCR-pMHC database. Figure 8B,C show the distribution of structures based on the length of the peptide found in the pMHC database and the TCR-pMHC database.

### Modelling the TCR-pMHC complex

The models produced by the TCRpMHCmodels method were generated using the automodel class from MODELLER v9.18^36^ with default settings. The automodel class takes two inputs: i) one or multiple template structures, and ii) an alignment of the target sequence and the sequence of the selected templates, in PIR format. In TCRpMHCmodels both the template selection and the alignment are generated automatically.

To calculate sequence identities, all sequences were aligned to the most similar HMM profile (either MHC, TCR α or TCR β) before calculating the sequence identity. These alignments were further used in the alignment file, after selecting the best templates. Using the structural templates and the alignment, MODELLER then builds a structural model of the target by optimally satisfying spatial restraints derived from the alignment.

### Template selection

#### Template selection for the pMHC

When modelling the pMHC, we investigated four different template selection methods: (i) OneUnweighted, (ii) OneWeighted, (iii) MultiWeighted and (iv) MultiUnweighted. In each of these template selection methods, we only used templates with the same peptide length. Using the in-house MHC class I HMM profile, we aligned the MHC chain of the target to templates found in the pMHC database (see pMHC database for further details about the class I HMM profile). The alignment was generated using hmmalign with the–trim option. After the alignment, we calculated the sequence identity between the target and each of the templates, excluding all insertions.

In the OneUnweighted method, the sequence identity was calculated by summing the identities from the peptide and the MHC alignment, dividing with sum of the peptide and HMM alignment lengths (excluding gaps). The templates were next sorted based on the sequence identity and the template with the highest sequence identity used for modelling.

The MultiUnweighted method uses the same approach to calculate the sequences identity, but instead of selecting only the single template with the highest identity score, this method selects multiple templates for model building. The selection of these multiple templates was done using a Hobohm1-like^58^ approach similarly to what we have described earlier^33^ by first sorting the templates according to sequence identity as described for method OneUnweighted. Next, the template with the highest sequence identity was selected and considered the best template. This template was always included. Next, looping through the sorted template list, additional templates were included if (1) they had an identity of less than 95% to any template already selected, and (2) their identity to the target was at least 80% of the identity of the top scoring best template.

In the OneWeighted method, the sequence identity measure was calculated by introducing a weight to the peptide and MHC sequence so that they each contributed equally to the sequence identity. Using the weighted sequence identity, we then selected the single template with the highest sequence identity.

In the MultiWeighted method, we used the weighted sequence identity, but selected multiple templates with the algorithm described above for the MultiUnweighted method.

#### Template selection for the TCR-pMHC

When modelling the TCR-pMHC, we investigated three different template selection methods, named: (i) OneUnweighted, (ii) OneWeighted and (iii) MultiWeighted. These three methods were performed as explained above, the only difference was the calculation of the weighted sequence identity. Here, the weighted sequence identity was calculated by introducing a weight to the peptide, MHC, TCR α and TCR β sequence so that the peptide and the MHC contributed to 1/3 of the sequence identity, while the TCR α and TCR β contributed to 1/6 of the sequence identity, respectively.

### Model validation

In order to assess the model accuracy of TCRpMHCmodels in situations where the structure is not known, we performed a leave-one-out (LOO) assessments of all structures in our template database. For each structure in the template database, we removed that structure from the database and built a structural model using the remaining templates. To further increase the model variability in terms of sequence identity of the adopted templates, we furthermore imposed four different template-target sequence identity thresholds of 99.9%, 95%, 90% and 80%, thereby removing any template having an identity higher than the selected threshold. For each structure in the template database, we therefore generated four different structural models imposing the four different sequence identity thresholds. The resulting models were then evaluated by comparing them to their native structure from the template database. This LOO assessments was used to evaluate the performance of both the pMHC and the final TCR-pMHC models.

### Random performance

In order to estimate a baseline performance value, we, for each structure in the template database, randomly selected another template from the database using the four different sequence identity thresholds as described above.

### TCRFlexDock

Protein-protein docking is a common method for assembling multi-chain proteins^59,60^. Thus, we compared TCRpMHCmodels with the CDRPep protocol from TCRFlexDock protocol^29^. TCRFlexDock is a specialised protein docking method for predicting the correct orientation between the TCR and pMHC molecules. While docking, the TCRFlexDock CDRPep protocol allows for some flexibility in the CDR loops and the MHC bound peptide. For each TCR-pMHC complex in the TCR-pMHC template database we modelled the TCR and the pMHC, and we then used the TCRFlexDock protocol to assemble these two models. As described in the TCRFlexDock protocol, we created 1000 docking decoys and each decoy was subsequently scored using ZRANK^39^. The decoy with the lowest ZRANK score was selected as the best TCR-pMHC complex from the protocol, and this complex was evaluated by comparing it to the native structure from the template database.

### Model performance

To assess the quality of the structural models, we used the root-mean-square deviation (RMSD) between the Cα atoms from the model and the Cα atoms in the native structure from the template database, after making a structural alignment of the model and the template. The structural alignment was made using the Superimposer class from BioPython^61^ minimising the distance between the Cα atoms in the model and the structural template before calculating the RMSD. To evaluate the model accuracy of the different parts in TCR-pMHC complex we generated four RMSD values, the TCR-pMHC RMSD, the TCR RMSD, the pMHC RMSD and the peptide RMSD. Each of these RMSD values were defined by calculating the RMSD after structural alignment of the different TCR-pMHC, the TCR, the pMHC and the peptide, respectively.

After superimposing, we used the template modelling score (TM-score), calculated as described by Y. Zhang *et al*.^62^ between all the Cα atoms. The TM-score is a length-independent metric used for measuring structural similarity between two proteins. The TM-score ranges between 0 and 1, where a TM-score of 1 indicates a perfect match between two structures. A TM-score below 0.2 corresponds to randomly choosing an unrelated protein and a TM-score higher than 0.5 assumes that the two structures roughly have the same structural fold.

To evaluate the model quality of the docking models from the TCRFlexDock protocol, we used the DockQ score calculated with the DockQ tool^40^. The DockQ score is made by combining Fnat, LRMS, and iRMS to a single score ranging between 0 and 1 that can be used to assess the quality of protein docking models.

## Supplementary information

Supplementary figures and table
