# Supplementary material for: TCRpMHCmodels: Structural modelling of TCR-pMHC class I complexes
Source: Sci Rep. 2019 Oct 10;9:14530. doi: 10.1038/s41598-019-50932-4 (PMC6787230; doi:10.1038/s41598-019-50932-4)
Supplement: Supplementary file 1 — Supplementary figures and table [file 41598_2019_50932_MOESM1_ESM.docx]

TCRpMHCmodels: Structural modelling of TCR-pMHC class I complexes

Kamilla Kjærgaard Jensen, Vasileios Rantos, Emma Jappe, Tobias Hegelund Olsen, Martin Closter Jespersen, Vanessa Jurtz, Leon Eyrich Jessen, Esteban Lanzarotti, Swapnil Mahajan, Bjoern Peters, Morten Nielsen, Paolo Marcatili

## Supplementary figures


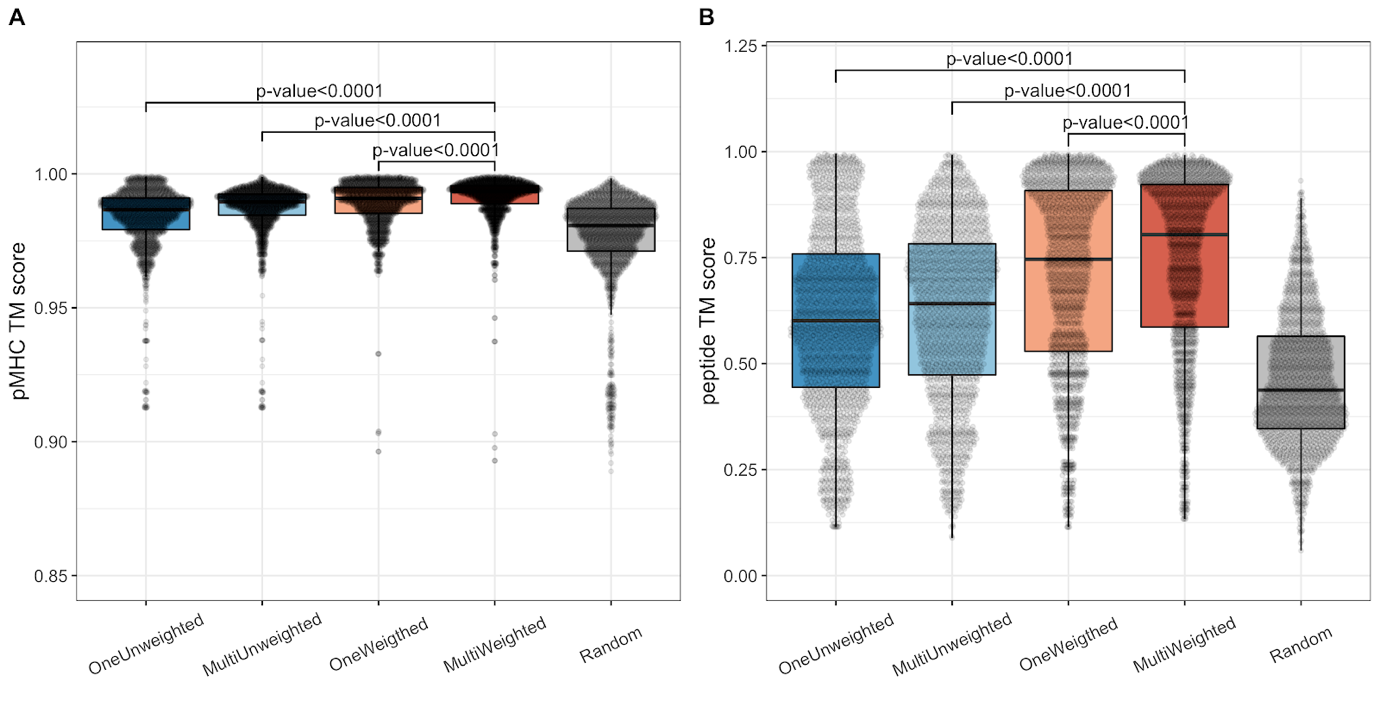


**Supplementary Figure S1:** TM-score performances for the different template selection methods. **A)** The TM-score performance for the pMHC complex. **B)** The TM-score for the peptide. For each target in the template database we generate four models using the four different sequence identity thresholds. Method OneUnweighted uses only a single template with a weighted sequence identity, while method MultiUnweighted uses multiple templates with a weighted sequence identity. Method OneWeighted used a single template and the weighted sequence identity. MultiWeighted uses multiple templates and the weighted sequence identity. The four different template selection methods are compared with a random baseline (see method for more details). Statistical comparison was performed using the Wilcoxon signed-rank test.

**
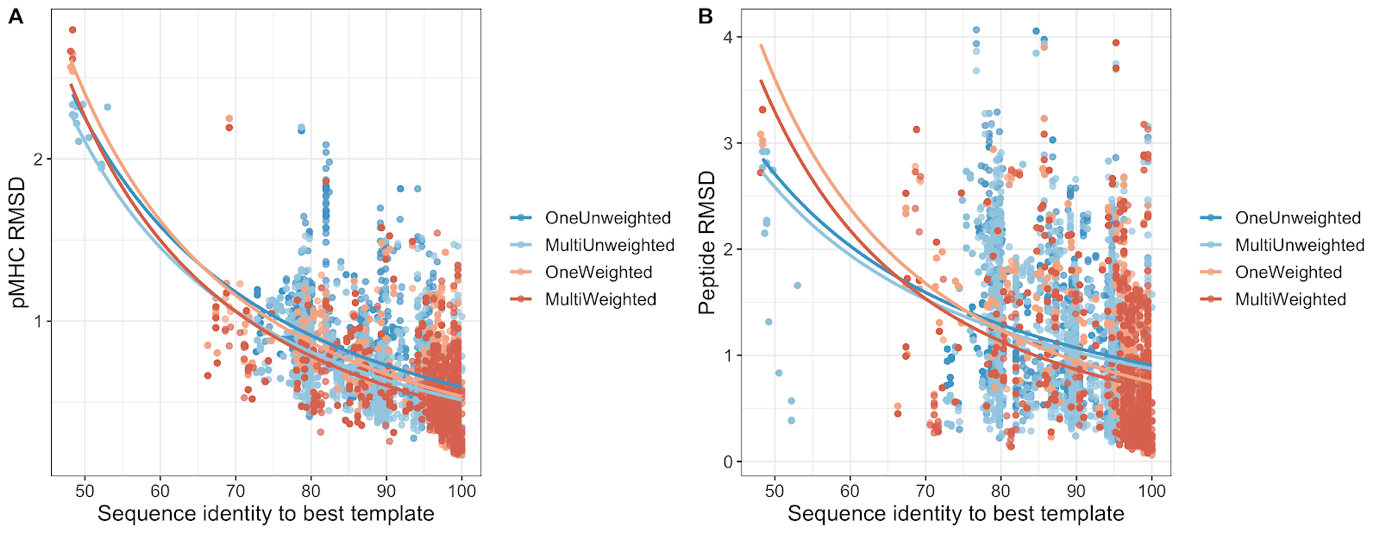
**

**Supplementary Figure S2:** Chothia-Lesk plot showing the RMSD performance for the pMHC models generated using the different template selection methods (see Method section). **A)** Shows the RMSD performance for the pMHC complex. **B)** Shows the RMSD performance for the peptide. The sequence identity to the best template is calculated using the unweighted sequence identity.


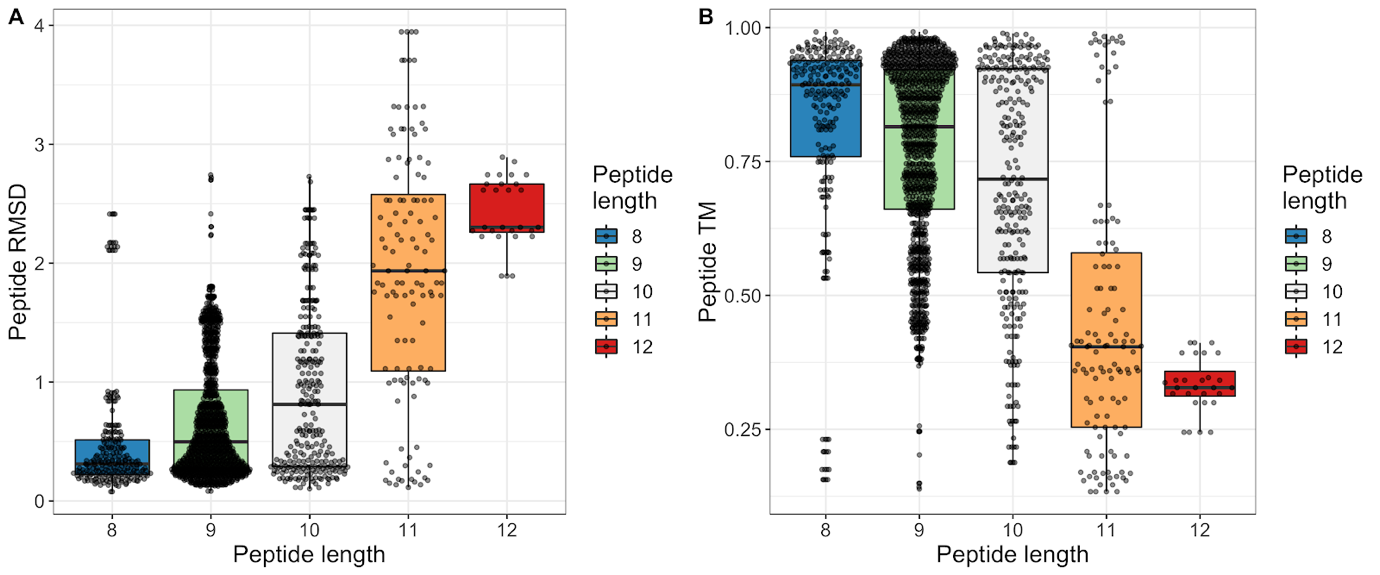


**Supplementary Figure S3:** **A)** Peptide RMSD performance for the pMHC models based on peptide length and **B)** the TM-score for the peptide for the pMHC models based on peptide length. Each pMHC model were produced using the MulitWeighted method for template selection.


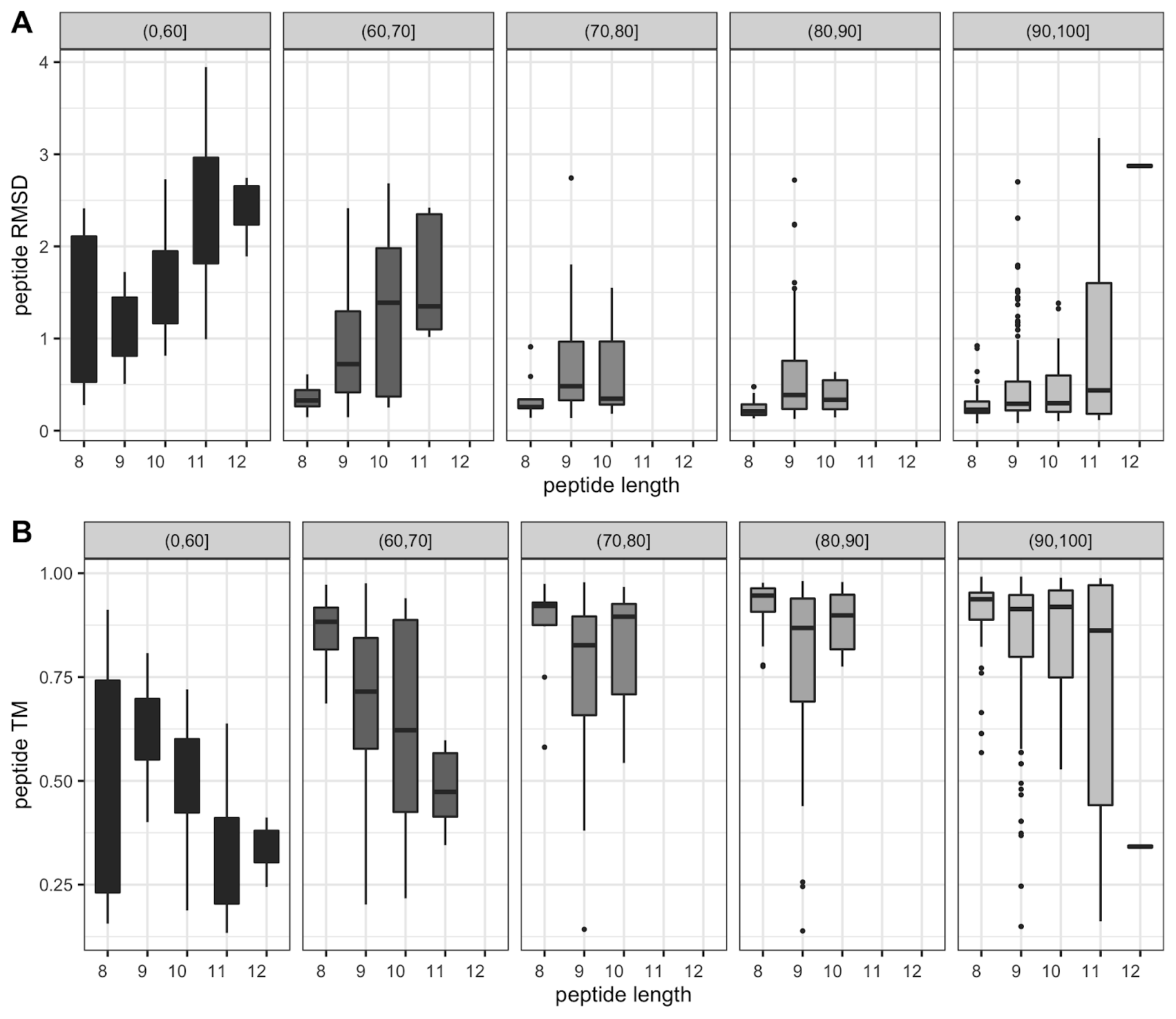


**Supplementary Figure S4:** **A)** Peptide RMSD accuracy for each pMHC model based on peptide length binned according to the sequence identity to the best template. **B)** TM-scores for the peptide for each pMHC model based on peptide length binned according to the sequence identity to the best template. The results shown in these plots are based on the pMHC models produced using the MulitWeighted method for template selection.


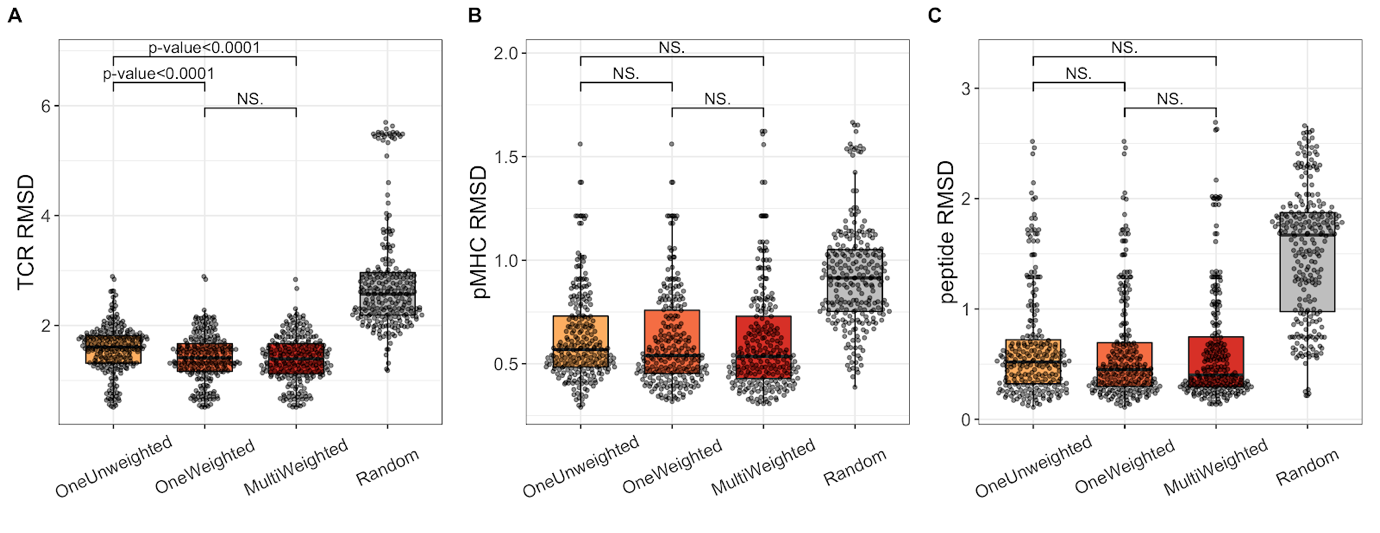


**Supplementary Figure S5:** The RMSD accuracy for the different template selection methods using **A)** the TCR RMSD, **B)** the pMHC RMSD and **C)** the peptide RMSD. For each target in the template database we generate four models using the four different sequence identity thresholds and evaluate the generated models using the RMSD for the TCR-pMHC complex. The OneUnweighted method uses only a single TCR-pMHC template with no weights on the sequence identity. The OneWeighted method uses only a single TCR-pMHC template and a weighted sequence identity. The MultiWeighted method uses the weighted sequence identity and multiple templates. The three different template selection methods are compared with a random baseline shown in grey. Statistical comparison was performed using the Wilcoxon signed-rank test.

**Supplementary Figure S6:** The TCR-pMHC TM-score accuracy for the different template selection methods. For each target in the TCR-pMHC template database we generate four models using the four different sequence identity thresholds and evaluate the generated models using the RMSD for the TCR-pMHC complex. The OneUnweighted method uses only a single TCR-pMHC template with no weights on the sequence identity. The OneWeighted method uses only a single TCR-pMHC template and a weighted sequence identity. The MultiWeighted method uses the weighted sequence identity and multiple templates. The three different template selection methods are compared with a random baseline shown in grey. Statistical comparison was performed using the Wilcoxon signed-rank test.


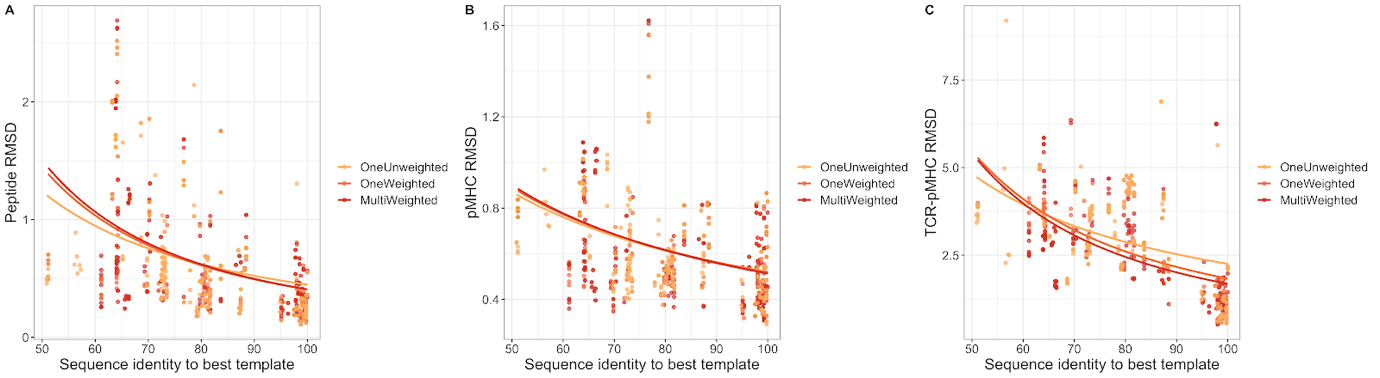
**Supplementary Figure S7:** Chothia-Lesk plot showing the RMSD accuracy for the TCR-pMHC models generated using the different template selection methods (see method section for more details). **A)** Shows the TCR-pMHC RMSD accuracy. **B)** Shows the pMHC RMSD accuracy. **C)** Shows the peptide RMSD accuracy. The sequence identity to the best template is calculated using the unweighted sequence identity.


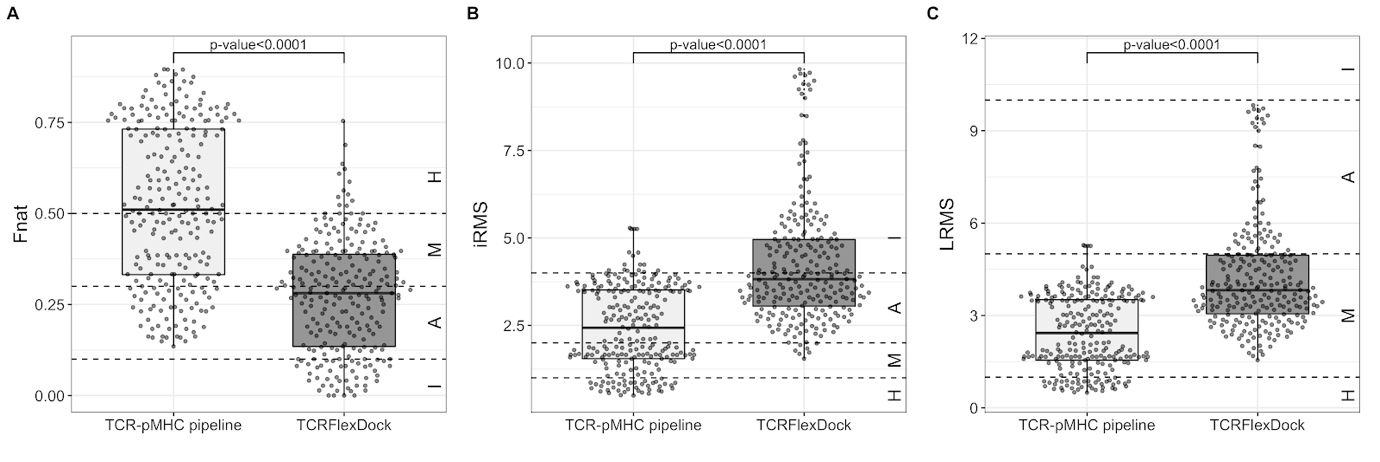


**Supplementary Figure S8:** Benchmark analysis of the TCR-pMHC models, showing different performance values between the models produced by TCRpMHCmodels and TCRFlexDock. **A)** Shows the Fnat accuracy **B)** Shows the iRMS accuracy and **C)** shows the LRMS accuracy. The statistical comparison was performed using the Wilcoxon signed-rank test and the dashed line indicates the thresholds for the four quality classes: High (H), Medium (M), Acceptable (A) and Incorrect (I) (see Method section).


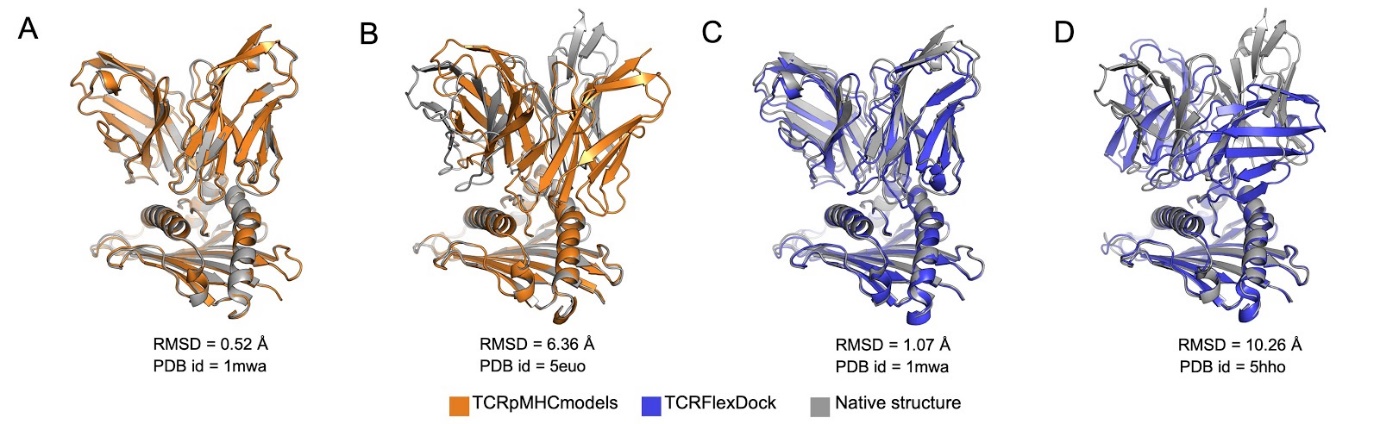


**Supplementary Figure S9:** Visualisation of high and low quality models generated by TCRpMHCmodels and TCRFlexDock. The orange and blue structures in the figure are models generated by TCRpMHCmodels and TCRFlexDock respectively, and native structures are shown in gray. To get a good view of the TCR orientation to the pMHC we superimposed only the pMHC, but the the RMSDs shown in the figure was calculated by superimposing all the C-alpha atoms in the TCR-pMHC model with all the C-alpha atoms in the native structure after which the RMSD was calculated for all C-alpha atoms. **A)** High quality model generated with TCRpMHCmodels, PDB id: 1mwa, RMSD: 0.52 and Fnat: 0.719. **B)** Low quality model generated with TCRpMHCmodels, PDB id: 5euo, RMSD: 6.36 and Fnat: 0.377. **C)** High quality model generated with TCRFlexDock, PDB id: 1mwa, RMSD: 1.07 and Fnat: 0.484. **D)** Low quality model generated with TCRFlexDock PDB id: 5hho, RMSD: 10.26 and Fnat: 0.020. Structural representations were made in PyMOL.


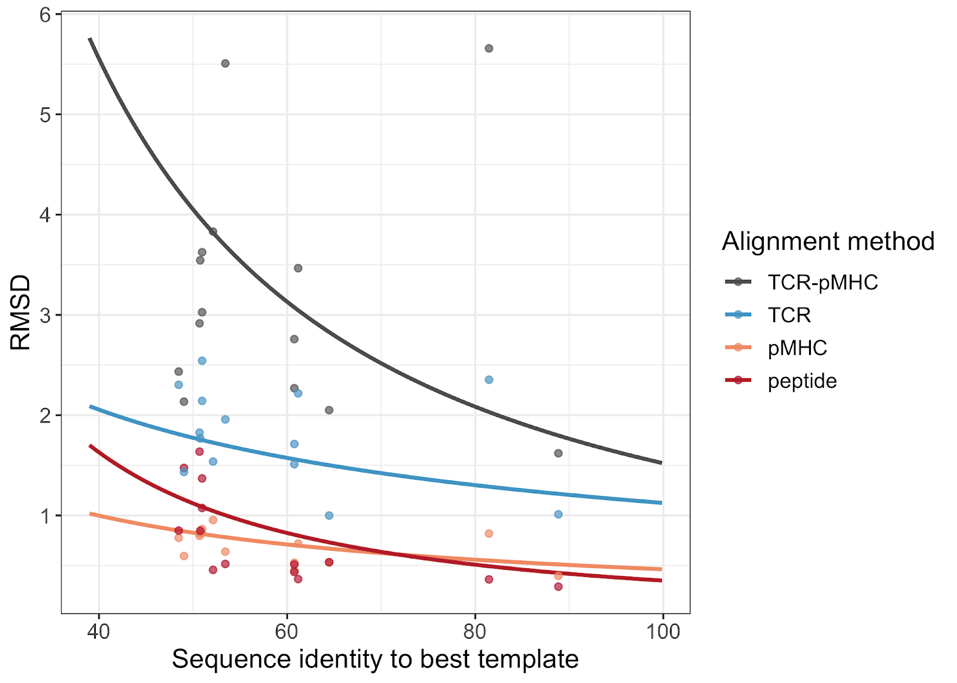


**Supplementary Figure S10:** The RMSD accuracy for the TCR-pMHC models generated using TCRpMHCmodels. The TCR-pMHC RMSD (grey), the TCR RMSD (blue), the pMHC RMSD (orange) and the peptide RMSD (read).


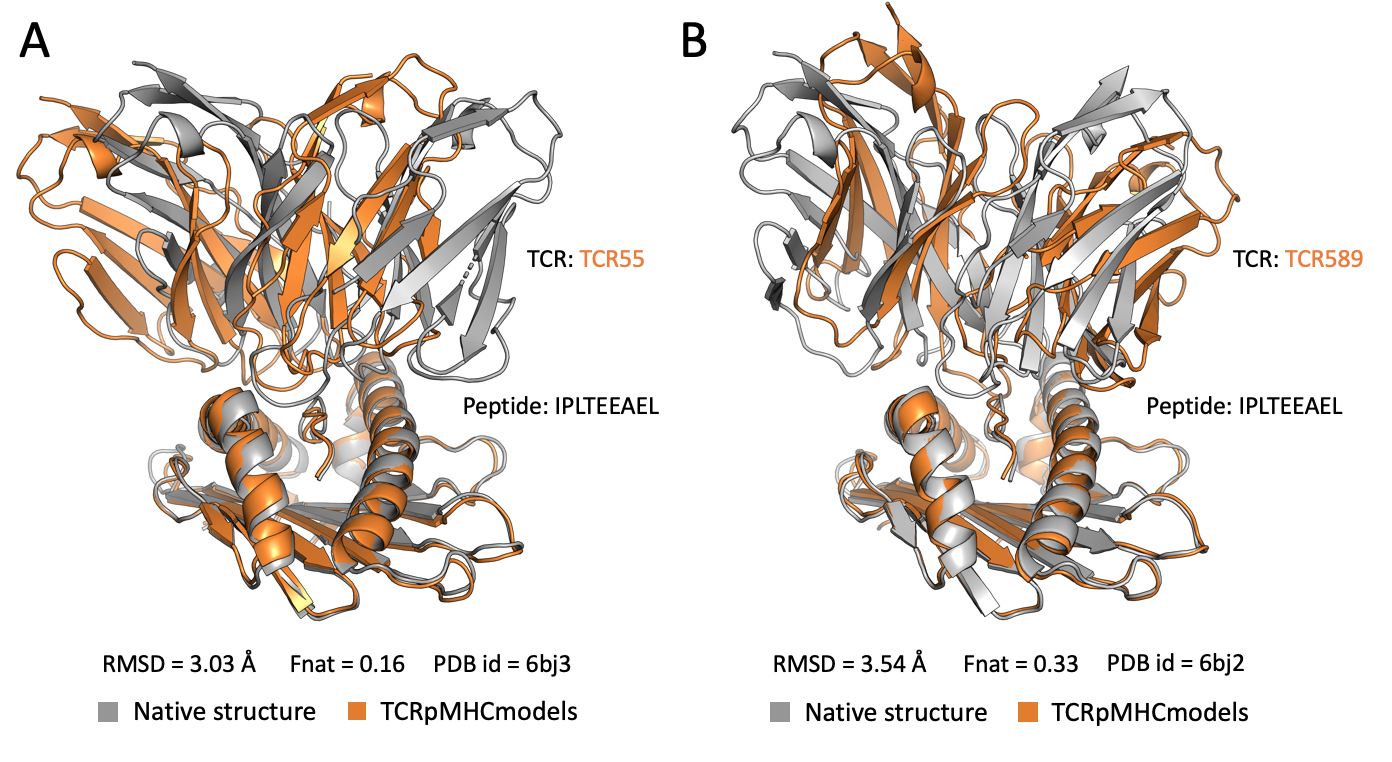


**Supplementary Figure S11:** Visualisation of a case where the same TCR binds different peptides. The native structures are shown in gray, while the models generated with TCRpMHCmodels are shown in orange. **A)** Shows the TCR-pMHC complex with the TCR55, PDB id: 6bj3. **B)** Shows the TCR-pMHC complex with the TCR589, PDB id: 6bj2. To get a good view of the TCR-pMHC models we superimposed only the pMHC, but the RMSDs shown in the figure was calculated by superimposing the C-alpha atoms in the TCR-pMHC model with the C-alpha atoms in the native structure. Structural representations were made in PyMOL.


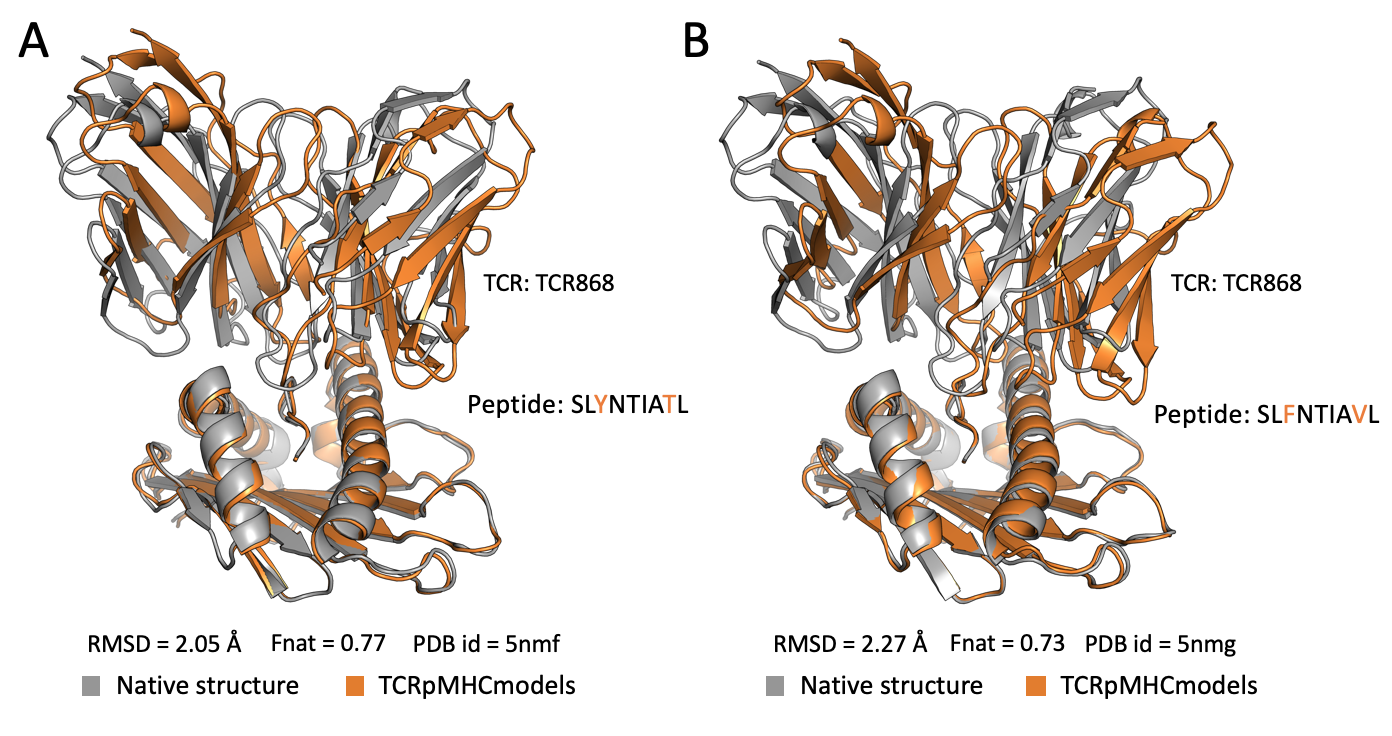


**Supplementary Figure S12:** Visualisation of a case where the same TCR binds different peptides. The native structures are shown in gray while the models generated with TCRpMHCmodels are shown in orange. **A)** Shows the TCR-pMHC complex with the SLYNTIATL peptide, PDB id: 5nmf. **B)** Shows the TCR-pMHC complex with the SLFNTIAVL peptide, PDB id: 5nmg. To get a good view of the TCR-pMHC models we superimposed only the pMHC, but the RMSDs shown in the figure was calculated by superimposing the C-alpha atoms in the TCR-pMHC model with the C-alpha atoms in the native structure. Structural representations were made in PyMOL.

## Supplementary tables

**Supplementary table S1:** The RMSD accuracy for the TCR-pMHC models generated using TCRpMHCmodels for the 14 TCR-pMHC structures not found in the TCR-pMHC database.

| **PDBid** | **TCR-pMHC rmsd** | **TCR rmsd** | **pMHC rmsd** | **Peptide rmsd** | **Sequence identity of best template** |
| --- | --- | --- | --- | --- | --- |
| 5isz | 1.62 | 1.01 | 0.40 | 0.29 | 88.84 |
| 5ivx | 5.51 | 1.96 | 0.64 | 0.52 | 53.43 |
| 5jzi | 3.47 | 2.22 | 0.72 | 0.37 | 61.17 |
| 5nme | 2.76 | 1.71 | 0.53 | 0.51 | 60.76 |
| 5nmf | 2.05 | 1.00 | 0.53 | 0.53 | 64.46 |
| 5nmg | 2.27 | 1.51 | 0.45 | 0.44 | 60.76 |
| 5tez | 5.66 | 2.35 | 0.82 | 0.36 | 81.46 |
| 5wkf | 2.14 | 1.43 | 0.60 | 1.47 | 49.03 |
| 5wkh | 2.92 | 1.83 | 0.80 | 1.64 | 50.69 |
| 5wlg | 3.83 | 1.54 | 0.95 | 0.46 | 52.12 |
| 5xot | 3.63 | 2.14 | 0.87 | 1.37 | 50.96 |
| 6bj2 | 3.54 | 1.77 | 0.82 | 0.85 | 50.76 |
| 6bj3 | 3.03 | 2.54 | 0.82 | 1.07 | 50.96 |
| 6bj8 | 2.43 | 2.30 | 0.78 | 0.85 | 48.47 |
| **Mean** | **3.20** | **1.81** | **0.69** | **0.77** | **58.85** |
